# The Clinical Landscape of Circulating Tumor DNA in Gastrointestinal Malignancies

**DOI:** 10.3389/fonc.2018.00263

**Published:** 2018-07-16

**Authors:** Kentaro Sawada, Daisuke Kotani, Hideaki Bando

**Affiliations:** ^1^Department of Gastroenterology and Gastrointestinal Oncology, National Cancer Center Hospital East, Kashiwa, Japan; ^2^Department of Gastroenterology, Hokkaido University Hospital, Sapporo, Japan

**Keywords:** circulating tumor DNA, colorectal cancer, gastric cancer, esophageal cancer, gastrointestinal malignancies

## Abstract

Technologies for genomic analyses have revealed more details in cancer biology and have changed standard treatments for cancer, including the introduction of targeted gene-specific therapy. Currently, liquid biopsies are increasingly being utilized in clinical trials and research settings to analyze circulating tumor DNA (ctDNA) from peripheral blood. Several studies have shown the potential of ctDNA in the screening, prognostication, molecular profiling, and monitoring of gastrointestinal malignancies. Although limitations continue to exist in the use of ctDNA, such as method standardization, the sensitivity, concordance with tumor tissue, and regulatory issues, this field offers promising benefits for cancer treatment. A deeper understanding of tumor biology *via* ctDNA analyses and ctDNA-guided clinical trials will lead to the increasing use of ctDNA in clinical practice in the near future; this development will result in the improvement of outcomes among patients with gastrointestinal malignancies.

## Introduction

Over several decades, the gold standard in the diagnosis and screening of tumors has been tissue biopsy (1). However, conventional tissue biopsies are invasive, painful, and carry a risk of complications such as bleeding, local infection, and damage to neighboring tissues. Moreover, a tissue biopsy cannot always reflect tumor dynamics or response to treatment. The recent era of precision medicine, which represents a paradigm shift in cancer, has challenged the gold standard in diagnosis by introducing a transition from tissue biopsy to liquid biopsy. Compared with tissue biopsies, liquid biopsies carry minimal potential risk and can be repeatedly performed in routine practice during cancer treatment by using peripheral blood. Furthermore, liquid biopsies have the potential to provide more complete information regarding the biology of whole tumors despite tumor heterogeneity. Liquid biopsies include the testing of soluble factors, such as circulating tumor DNA (ctDNA) and circulating cell-free DNA (cfDNA), as well as proteins and tumor markers (2). cfDNA is highly fragmented DNA that is released from necrotic or apoptotic cells into the bloodstream (3–5). cfDNA consists of DNA from healthy cells and tumor cells, whereas ctDNA is defined as DNA that is derived only from primary or metastatic tumor cells.

Since Mandel and Metais (6) reported fragmented DNA in blood for the first time in 1948, technologies for cfDNA quantification have changed over 70 years from quantitative polymerase chain reaction to complex BEAMing and deep next-generation sequencing (NGS), thus achieving improvements in the sensitivity and specificity of cfDNA detection. With the development of sensitive techniques that can detect rare mutations, the heterogeneous landscape of tumors can be determined using blood samples. In fact, National Comprehensive Cancer Network guideline for non-small cell lung cancer (version 4. 2018) states that plasma biopsy should be considered if repeat biopsy is not feasible (7).

Here, we review ctDNA in gastrointestinal malignancies by focusing on clinical utility and future perspectives.

## ctDNA and Related Technologies

The presence of cfDNA in the blood is a well-established fact, and DNA fragments are released from dying cells because of cellular turnover or other types of cell death (2). In cancer patients, a fraction of cfDNA is tumor derived and is termed ctDNA. ctDNA originates from primary tumors, metastatic tumor cells, or circulating tumor cells. ctDNA molecules are shorter than non-mutant cfDNA molecules in plasma, as demonstrated by PCR (8, 9) and sequencing (10, 11).

Representative approaches for analyzing ctDNA are summarized in Table 1 (12). Mutation-specific real-time or endpoint PCR has been used for the detection of point mutations in ctDNA (13–17). More recently, digital PCR methods such as BEAMing and droplet digital PCR have been developed to improve the identification of genomic alterations in ctDNA (18–20). The recent implementation of NGS has allowed the direct sequence-based detection of chromosomal alterations in plasma DNA (21–23); however, it is necessary to distinguish the relatively few somatic alterations in ctDNA from the larger numbers of structural variants present in the germline cells of all individuals. Bioinformatics-based filters that enrich high-confidence somatic structural alterations while eliminating germline and artifactual changes have been developed (12). In addition, importantly, amplification in ctDNA can be depend on both the amount of ctDNA in the plasma due to high tumor burden and high copy number of specific gene. Commercially available kits for the NGS assays of ctDNA are summarized in Table 2.

**Table 1:** Available assays of detection of circulating tumor DNA (12, 24).

Characteristic	PCR assays		Next-generation sequencing (NGS) assays	
	Allele-specific PCR	Emulsion PCR	Amplicon-based targeted NGS	Capture-based targeted NGS
Variants potentially detected	Known recurring mutations	Known recurring mutations	Any exonic mutations, copy number gains	Exonic mutations, intronic gene fusions, copy number gains

Quantitation	Semiquantitative	Absolute or relative quantitation, wide dynamic range	Quantitation of relative AF, but vulnerable to PCR amplification bias	Quantitation of relative AF

Speed	Rapid	Rapid	Slower	Slower

Examples	Cobas (Roche), therascreen (Qiagen)	Droplet digital PCR (Biorad), BEAMing (Sysmex Inostics)	Tam-seq (Inivata)	Guardant360 (Guardant), cancerselect (personal genome diagnostics)

**Table 2:** Commercially available circulating tumor DNA next-generation sequencing assays.

Panel	Company	Gene number	Assays
Guardant360	Guardant Health	73	Capture-based
PlasmaSELECT-R64	Personal Genome Diagnostics	64 + MSI	Capture-based
FoundationACT	Foundation Medicine	62	Capture-based
Oncomine Colon cfDNA Assay	Thermo Fisher Scientific	14	Amplicon-based

## Early Detection of Cancer

The early detection of cancer is one of the most important issues in reducing cancer-related deaths. In many cases, gastrointestinal cancer is detected *via* endoscopy or CT scans conducted for symptoms such as anorexia, abdominal pain, or constipation. ctDNA may have a potential role in the noninvasive early diagnosis and screening of gastrointestinal cancer. Even localized cancers shed DNA into circulation; therefore, ctDNA can be detected in patients with localized cancers, in addition to patients with advanced or metastatic cancers.

In a study across several early and late-stage cancers, ctDNA was detected in 73, 57, and 48% of patients with colorectal cancer (CRC), gastroesophageal cancer, and pancreatic cancer, respectively (25). The use of several biomarkers in ctDNA including the levels of overall ctDNA, *ALU247* fragment concentration (26), *KRAS* mutations (27, 28), *TP53* mutations (29, 30), *BRAF* mutations (28), and *septin 9* (*SEPT9*) methylation (31–34) have been demonstrated for the diagnosis of CRC. Also, detection of methylated SEPT9 DNA in plasma is US FDA approved as a blood test for CRC screening. Compared with biomarkers for CRC, biomarkers for the diagnosis of gastric cancer (GC) and esophageal cancer have been assessed in a relatively small number of cohorts (25, 35). For the early detection or screening of cancers including CRC, GC, and esophageal cancer, the sensitivity of ctDNA analysis needs to be improved. Analysis that can be performed using a few milliliters of blood would be suitable for cancer screening; however, increasing the analytical sensitivity beyond 0.1% may not provide clinical benefits because it also leads to difficulties in distinguishing oncological mutations and sampling noise. In fact, cancer-associated genomic alterations have been found in plasma from healthy individuals (36). In addition, because many cancers share common gene mutations such as *TP53* mutations and *KRAS* mutations, ctDNA presents challenges in the detection of the specific organ sites of malignancies. To overcome these issues in ctDNA, the methylation profiling of cfDNA has been investigated in cancer diagnosis. Methylation haplotyping in plasma is a promising strategy for the early detection of a tumor and its primary growth site (37). Studies have reported the utility of methylation scores from over 9,000 CpG sites in cfDNA for cancer detection, with 76.3% accuracy for the prediction of cancer type (38).

**Figure 1:**
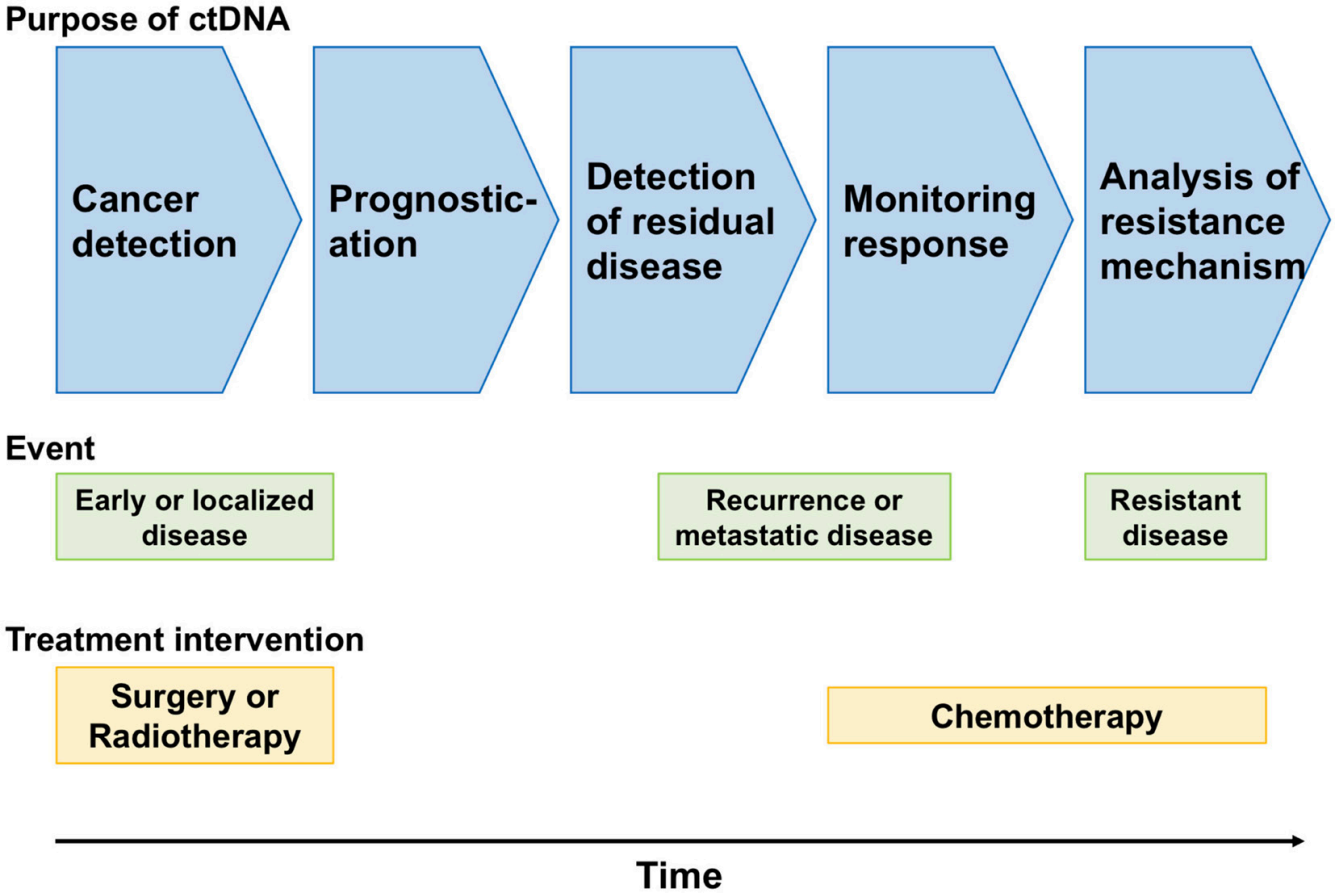
Clinical applications of circulating tumor DNA (ctDNA) in gastrointestinal malignancies.

Despite the above hurdles to the use of ctDNA in cancer screening, it is expected that the clinical use of ctDNA is less than a decade away because of its utility and convenience in cancer screening (Figure 1).

## Prognosis and Detection of Residual Disease

Following curative therapy for gastrointestinal malignancies, ctDNA may be a potential biomarker for minimal residual disease. The detection of ctDNA even in the absence of any other clinical evidence of disease may mean that the patient has higher risk of relapse. In a cohort of 230 patients with stage II CRC, the assessment of ctDNA using the Safe-SeqS NGS method at the first visit after surgery indicated that recurrence-free survival at 3 years was 0% in a ctDNA-positive group and 90% in a ctDNA-negative group (39). Other studies have also demonstrated that the persistent detection of ctDNA after local therapy (surgery or radical radiotherapy) predicts a high risk of relapse in patients with colon cancer (40, 41). In addition, methylated *BCAT1*/*IKZF1* have been evaluated as biomarkers for CRC (42, 43). Of 397 patients with CRC who underwent primary tumor resection, odds ratio of a positive CEA test for recurrence was 6.9 (95% CI 2–22) compared to 14.4 (5–23, 25–40) for *BCAT1*/*IKZF1*.

In a meta-analysis of 16 studies including 1,193 patients with GC, the presence of ctDNA was significantly associated with the shorter disease-free survival (HR 4.36, 95% CI 3.08–6.16, *p* < 0.001) and overall survival (HR 1.77, 95% CI 1.38–2.28, *p* < 0.001) of GC patients, with high specificity (0.95, 95% CI 0.93–0.96) and relatively moderate sensitivity (0.62, 95% CI 0.59–0.65) (44). Another study demonstrated that the level of ctDNA was associated with tumor recurrence in patients who underwent curative surgery for GC (45). Similarly, several studies have reported the tumor-associated mutations in ctDNA and the prognosis of patients with esophageal cancer (46, 47); however, these studies included a limited number of patients, and further investigations are warranted. Almost all of these studies followed a retrospective design and provided limited validation for clinical use in gastrointestinal malignancies. One of the ideal applications of ctDNA is in the early detection of residual disease or recurrence compared with CT imaging and tumor markers. A more attractive idea is patient-specific ctDNA panels in patients who have undergone curative surgery (41, 48). Individual surgical tumor samples may provide a great opportunity to obtain tumor DNA from each patient to guide the design of patient-specific ctDNA panels from peripheral blood samples. Despite hurdles such as tumor heterogeneity, validation, regulatory issues, and quality control of individual panels, patient-specific ctDNA panels are potential biomarkers for postoperative monitoring.

## Biomarkers of Chemotherapy Response and Resistance in Metastatic Disease

Another clinical potential of ctDNA is in the determination of systemic chemotherapy regimens, the prediction of response to chemotherapy, and the identification of resistance mechanisms. The short half-life of ctDNA enables the real-time monitoring of cancer burden, in contrast to radiological imaging or tumor markers. Indeed, a prospective study of 53 patients with metastatic CRC demonstrated that early changes in ctDNA during first-line chemotherapy predicted later radiologic response. Significant reductions in ctDNA levels were observed before cycle 2 and were correlated with CT response at 8–10 weeks (odds ratio 5.25 with a 10-fold ctDNA reduction; *p* = 0.016) (49). Blood-based monitoring is an ideal strategy during cancer treatment because of its minimal invasiveness and avoidance of radiation exposure.

### RAS Mutations in Metastatic CRC

The assessment of *RAS* status has been mandatory in patients with metastatic CRC to predict the response of cetuximab and panitumumab to anti-EGFR antibodies (50–55). A double-blinded prospective study of 106 patients with mCRC has been performed to compare the *KRAS* mutation status assessed using tumor tissue *via* routine gold-standard methods to that assessed using plasma DNA *via* qPCR-based methods; the resultant specificity and sensitivity for the detection of *KRAS* point mutations were 98 and 92%, respectively, resulting in 96% concordance (28). In addition, the retrospective exploratory analysis in a biomarker subgroup of the CORRECT trial, which was a phase III trial investigating the efficacy and safety of regorafenib in patients with mCRC, confirmed the utility of detecting *KRAS, BRAF*, and *PIK3CA* mutations in ctDNA. Plasma DNA detected with BEAMing in 503 patients demonstrated that mutation status in ctDNA changed dynamically during chemotherapy and differed from that in pretreatment archival tissue (56). Currently, the OncoBEAM RAS CRC assay is the only European committee *in vitro* diagnostic test for *RAS* mutations in ctDNA. This assay is a qualitative PCR-based test and allows for the detection of 34 mutations within exons 2, 3, and 4 of *KRAS* and *NRAS* genes from a single blood sample. Four large cohort studies have been reported to achieve high concordance of approximately >90% (range, 89.7–93.3%) between OncoBEAM using plasma ctDNA and tumor tissue analysis in patients with CRC (57–60).

Acquired resistance to anti-EGFR antibody therapy has also been found by using ctDNA analyses. ctDNA from 28 patients receiving panitumumab monotherapy was assessed using qPCR, and 9 out of 24 (38%) patients whose tumors were initially *KRAS* wild-type showed *KRAS* mutations in ctDNA after panitumumab treatment (61). This study suggested that the emergence of *KRAS* mutations is a mechanism of resistance to anti-EGFR therapy and that these mutations may be detected in ctDNA as a more sensitive monitoring tool than radiological imaging. More recently, other studies have also demonstrated mutations associated with the resistance and decline of mutant *KRAS* clones after the withdrawal of anti-EGFR therapy (62, 63).

### BRAF Mutations in Metastatic CRC

*BRAF* mutations have been confirmed to be associated with poor prognosis in patients with metastatic CRC; moreover, the limited efficacy of anti-EGFR therapy in patients with *BRAF*-mutant metastatic CRC has been shown in several studies (64–66). The analysis of *BRAF* mutations in ctDNA by using qPCR-based methods has shown specificity and sensitivity of 100% (28). Based on preclinical studies (67, 68), the clinical trials of dual EGFR and MAPK signaling pathway inhibition in patients with *BRAF*-mutant metastatic CRC is ongoing. In a phase Ib study of a combination therapy of dabrafenib, trametinib, and panitumumab, *BRAF* V600E mutant burden in ctDNA was more markedly reduced in responders than in nonresponders, and the emergence of *RAS* mutations was seen with disease progression in 9 of 22 patients (41%) (69). This exploratory analysis suggested that the monitoring of *BRAF* V600E mutant fraction in ctDNA could effectively predict response to combination therapy including a BRAF inhibitor and that overcoming the emergence of *RAS*-mutant subclones is important in combating resistance to this combination therapy.

### Other Alterations in Metastatic CRC

*HER2* or *MET* amplification is also known as a mechanism of resistance to anti-EGFR therapy in patients with metastatic CRC. The patient-derived xenograft models of *HER2*-amplified CRC showed resistance to anti-EGFR therapy (70, 71). In addition, the frequency of *HER2* amplifications increased from approximately 3% in treatment-naïve patients to over 10% in patients who were administered anti-EGFR therapy (72). Although there are few studies regarding the concordance of *HER2* status between ctDNA and tissue samples, 4 of 18 (22%) patients exhibited *HER2* amplification in ctDNA by digital PCR after cetuximab therapy despite being negative for *HER2* amplification prior to anti-EGFR therapy (73). The promising results of trastuzumab and T-DM1 combination therapy in the HERACLES trial (71) have encouraged clinical trials in patients with HER2-positive metastatic CRC such as the MyPathway trial (74) and the TRIUMPH trial (75); notably, the TRIUMPH trial includes patients with *HER2* amplification detected using not only tissue samples but also ctDNA analysis using an NGS-based method.

Another important alteration that causes resistance to anti-EGFR therapy is *MET* amplification. A preclinical model of *MET*-amplified CRC also showed resistance to anti-EGFR therapy (76). In fact, *MET* amplification in ctDNA was detected using NGS in 12 of 53 (22.6%) patients who showed disease progression with anti-EGFR therapy; no such amplification was detected in patients before cetuximab therapy. Furthermore, *MET* amplification in ctDNA was not detected in patients with *RAS* mutations after cetuximab therapy, thus suggesting that *MET* amplification is one of the mechanisms (other than *RAS* mutations) that cause resistance to anti-EGFR therapy (77). In a phase Ib trial of cabozantinib and panitumumab combination therapy, the preliminary evidence of efficacy in patients with MET-amplified metastatic CRC was reported (78).

### HER2 Amplification in GC

The amplification of the *HER2* gene or overexpression of the HER2 protein, which contributes to cancer progression, has been reported in approximately 20% of patients with advanced GC (79, 80). According to the results of the ToGA trial, HER2 is a key biomarker of HER2-targeted therapy using trastuzumab for advanced GC (79). The gold-standard diagnostic method for detecting HER2 positivity and suitability for trastuzumab therapy is an immunohistochemistry score of 3+ or 2+ with a positive result in fluorescence *in situ* hybridization. A retrospective study of 52 patients with advanced GC and 40 healthy volunteers demonstrated that the plasma *HER2*–*RPPH1* ratio (with *RPPH1* as a reference gene) was significantly higher in patients with HER2-positive tumors than those with HER2-negative tumors (81). More recently, the droplet digital PCR of *HER2* copy number in ctDNA has been reported. In a study of 60 patients with GC, including 17 patients who developed recurrence and 30 healthy volunteers, preoperative plasma *HER2* ratio correlated with tumor HER2 status; postoperative plasma *HER2* ratios were high during the recurrence of tumors, which were diagnosed as HER2-negative tumors in surgery samples (82). Considering that HER2 status may be altered after recurrence, the *HER2* copy number analysis in ctDNA enables the real-time evaluation of HER2 status and leads to more effective treatment choices with HER2-targeted agents.

## Future Perspectives

Overall, the data generated in all studies discussed above support the potential role of ctDNA in the diagnosis and treatment of patients with gastrointestinal malignancies. Despite a few limitations, including the standardization of detection and ctDNA quantification, the sensitivity, and concordance between ctDNA and tissue biopsies that currently hinder the routine use of ctDNA in clinical trials and clinical practice, its use would allow a deeper understanding of cancer biology and enable better cancer treatment, thus leading to improvements in patient survival.

In the context of clinical trials for metastatic disease treatment, several studies are ongoing or have been conducted using eligibility criteria based on gene alterations in ctDNA. A prospective study on the comprehensive ctDNA-guided treatment of advanced GC and lung cancers is ongoing in Korea (83). Another trial called the Targeted Agent and Profiling Utilization Registry, which is a large basket/umbrella trial sponsored by the American Society of Clinical Oncology, is accepting patient selection on the basis of ctDNA analysis (NCT02693535). In addition, an umbrella trial in patients with mCRC based on the molecular profiling of ctDNA, including the status of *HER2, BRAF* V600E, *BRAF* non-V600E, *MET*, or high tumor mutation burden, is ongoing in Japan. If promising results are obtained in these clinical trials, ctDNA will be used in routine clinical practice and in clinical trials in the near future.

Economic and regulatory issues still hinder the practical use of ctDNA. Although most guidelines recommend that comprehensive molecular profiling should be conducted, the substantial costs of NGS assays lead many community oncologists to rely on PCR tissue tests and do not understand the added benefit of a comprehensive genomic test. In addition, emerging ctDNA-guided clinical trials are essential to obtain approval for the use of ctDNA in clinical practice. These barriers need to be challenged, perhaps initially in patients with CRC, which is one of the most prevalent gastrointestinal malignancies worldwide. Simultaneously, further studies are needed on other gastrointestinal malignancies such as esophageal cancer and GC to identify the best gene biomarkers that are detectable in ctDNA for the diagnosis, prognosis, and prediction of therapy response.

## Concluding Remarks

The potential role of ctDNA in gastrointestinal malignancies has been shown in basic studies, retrospective studies, and limited prospective studies. A paradigm shift in cancer diagnosis and treatment in ctDNA-based clinical trials and clinical practice will occur in the near future, thus leading to the availability of more DNA sequence information compared with that in the past decade. Although some limitations continue to exist on the use of ctDNA in clinical practice and clinical trials, ctDNA-based personalized therapy promises to improve patient outcomes and quality of life.

## Author Contributions

This review was drafted by KS and DK and was revised by HB.

## Conflict of Interest Statement

The authors declare that the research was conducted in the absence of any commercial or financial relationships that could be construed as a potential conflict of interest. The handling Editor declared a past co-authorship with one of the authors [DK].

## References

[1] Wright JR Charles ES Emile K The first cancer biopsy. Int J Surg (2013) 11:106–7. 10.1016/j.ijsu.2012.11.017 23220086

[2] Siravegna G Marsoni S Siena S Bardelli A Integrating liquid biopsies into management of cancer. Nat Rev Clin Oncol (2017) 14:531–48. 10.1038/nrclinonc.2017.14 28252003

[3] Lecomte T Ceze N Dorval E Laurent PP Circulating free tumor DNA and colorectal cancer. Gastroenterol Clin Biol (2010) 34:662–81. 10.1016/j.gcb.2009.04.015 20832215

[4] Alix PC Schwarzenbach H Pantel K. Circulating tumor cells and circulating tumor DNA. Annu Rev Med (2012) 63:199–215. 10.1146/annurev-med-062310-094219 22053740

[5] Heitzer E Ulz P Geigl JB. Circulating tumor DNA as a liquid biopsy for cancer. Clin Chem (2015) 61:112–23. 10.1373/clinchem.2014.222679 25388429

[6] Mandel P Metais P Les acides nucleiques du plasma sanguin chezl’homme. C R Seances Soc Biol Fil (1948) 142:241–3. 18875018

[7] NCCN Clinical Practice Guidelines in Oncology (NCCN Guidelines) Colon Cancer Version 2. Available from: https://www.nccn.org/professionals/physician_gls/recently_updated.aspx (Accessed: May 31, 2018).

[8] Mouliere F Robert B Peyrotte EA Del Rio M Ychou M Molina F High fragmentation characterizes tumour-derived circulating DNA. PLoS One (2011) 6:e23418. 10.1371/journal.pone.0023418 21909401 PMC3167805

[9] Mouliere F El Messaoudi S Gongora C Guedj AS Robert B Del Rio M Circulating cell-free DNA from colorectal cancer patients may reveal high KRAS or BRAF mutation load. Transl Oncol (2013) 6:319–28. 10.1593/tlo.12445 23730412 PMC3660801

[10] Sun K Jiang P Chan KA Wong J Cheng YK Liang RH Plasma DNA tissue mapping by genomewide methylation sequencing for noninvasive prenatal, cancer, and transplantation assessments. Proc Natl Acad Sci U S A (2015) 112:E5503–12. 10.1073/pnas.1508736112 26392541 PMC4603482

[11] Underhill HR Kitzman JO Hellwig S Welker NC Daza R Baker DN Fragment length of circulating tumor DNA. PLoS Genet (2016) 12:e1006162 10.1371/journal.pgen.1006162 27428049 PMC4948782

[12] Haber DA Valculescu VE. Blood-based analyses of cancer: circulating tumor cells and circulating tumor DNA. Cancer Discov (2014) 6:650–61. 10.1158/2159-8290.CD-13-1014 24801577 PMC4433544

[13] Board RE Wardley AM Dixon JM Armstrong AC Howell S Renshaw L Detection of PIK3CA mutations in circulating free DNA in patients with breast cancer. Breast Cancer Res Treat (2010) 120:461–7. 10.1007/s10549-010-0747-9 20107891

[14] Wang JY Hsieh JS Chang MY Huang TJ Chen FM Cheng TL Molecular detection of APC, K-ras, and p53 mutations in the serum of colorectal cancer patients as circulating biomarkers. World J Surg (2004) 28:721–6. 10.1007/s00268-004-7366-8 15185002

[15] Yamada T Nakamori S Ohzato H Oshima S Aoki T Higaki N Detection of K-ras gene mutations in plasma DNA of patients with pancreatic adenocarcinoma: correlation with clinicopathological features. Clin Cancer Res (1998) 4:1527–32. 9626473

[16] Perkins G Yap TA Pope L Cassidy AM Dukes JP Riisnaes R Multi-purpose utility of circulating plasma DNA testing in patients with advanced cancers. PLoS One (2012) 7:e47020. 10.1371/journal.pone.0047020 23144797 PMC3492590

[17] Shi J Liu Q Sommer SS. Detection of ultrarare somatic mutation in the human TP53 gene by bidirectional pyrophosphorolysis-activated polymerization allele-specific amplification. Hum Mutat (2007) 28:131–6. 10.1002/humu.20423 17041903

[18] Dressman D Yan H Traverso G Kinzler KW Vogelstein B Transforming single DNA molecules into fluorescent magnetic particles for detection and enumeration of genetic variants. Proc Natl Acad Sci U S A (2003) 100:8817–22. 10.1073/pnas.1133470100 12857956 PMC166396

[19] Hindson BJ Ness KD Masquelier DA Belgrader P Heredia NJ Makarewics AJ High-throughput droplet digital PCR system for absolute quantitation of DNA copy number. Anal Chem (2011) 83:8604–10. 10.1021/ac202028g 22035192 PMC3216358

[20] Pekin D Skhiri Y Baret JC Le Corre D Mazutis L Salem CB Quantitative and sensitive detection of rare mutations using droplet-based microfluidics. Lab Chip (2011) 11:2156–66. 10.1039/c1lc20128j 21594292

[21] Leary RJ Sausen M Kinde I Papadopoulos N Carpten JD Craig D Detection of chromosomal alterations in the circulation of cancer patients with whole-genome sequencing. Sci Transl Med (2012) 4:162ra54. 10.1126/scitranslmed.3004742 23197571 PMC3641759

[22] Chan KC Jiang P Zheng YW Liao GJ Sun H Wong J Cancer genome scanning in plasma: detection of tumor-associated copy number aberrations, single-nucleotide variants, and tumoral heterogeneity by massively parallel sequencing. Clin Chem (2012) 59:211–24. 10.1373/clinchem.2012.196014 23065472

[23] Heitzer E Ulz P Belic J Gutschi S Quehenberger F Fischereder K Tumor-associated copy number changes in the circulation of patients with prostate cancer identified through whole-genome sequencing. Genome Med (2013) 5:30. 10.1186/gm434 23561577 PMC3707016

[24] Oxnard GR Paweletz CP Sholl LM Genomic analysis of plasma cell-free DNA in patients with cancer. JAMA Oncol (2017) 3:740–1. 10.1001/jamaoncol.2016.2835 27541382

[25] Bettegowda C Sausen M Leary RJ Kinde I Wang Y Agrawal N Detection of circulating tumor DNA in early- and late-stage human malignancies. Sci Transl Med (2014) 6:224ra24. 10.1126/scitranslmed.3007094 24553385 PMC4017867

[26] da Silva Filho BF Gurgel AP Neto MÁ de Azevedo DA de Freitas AC Neto JD Circulating cell-free DNA in serum as a biomarker of colorectal cancer. J Clin Pathol (2013) 66:775–8. 10.1136/jclinpath-2013-201521 23833048

[27] Lecomte T Berger A Zinzindohoué F Micard S Landi B Blons H Detection of free-circulating tumor-associated DNA in plasma of colorectal cancer patients and its association with prognosis. Int J Cancer (2002) 100:542–8. 10.1002/ijc.10526 12124803

[28] Thierry AR Mouliere F El Messaoudi S Mollevi C Lopez-Crapez E Rolet F Clinical validation of the detection of KRAS and BRAF mutations from circulating tumor DNA. Nat Med (2014) 20:430–5. 10.1038/nm.3511 24658074

[29] Baker SJ Preisinger AC Jessup JM Paraskeva C Markowitz S Willson JK p53 gene mutations occur in combination with 17p allelic deletions as late events in colorectal tumorigenesis. Cancer Res (1990) 50:7717–22. 2253215

[30] Fearon ER Vogelstein B A genetic model for colorectal tumorigenesis. Cell (1990) 61:759–67. 10.1016/0092-8674(90)90186-I 2188735

[31] Tóth K Sipos F Kalmár A Patai ÁV Wichmann B Stoehr R Detection of methylated SEPT9 in plasma is a reliable screening method for both left- and right-sided colon cancers. PLoS One (2012) 7:e46000. 10.1371/journal.pone.0046000 23049919 PMC3457959

[32] Tetzner R Model F Weiss G Schuster M Distler J Steiger KV Circulating methylated SEPT9 DNA in plasma is a biomarker for colorectal cancer. Clin Chem (2009) 55:1337–46. 10.1373/clinchem.2008.115808 19406918

[33] Grützmann R Molnar B Pilarsky C Habermann JK Schlag PM Saeger HD Sensitive detection of colorectal cancer in peripheral blood by septin 9 DNA methylation assay. PLoS One (2008) 3:e3759. 10.1371/journal.pone.0003759 19018278 PMC2582436

[34] Lofton-Day C Model F DeVos T Tetzner R Distler J Schuster M DNA methylation biomarkers for blood-based colorectal cancer screening. Clin Chem (2008) 54:414–23. 10.1373/clinchem.2007.095992 18089654

[35] Zhai R Zhao Y Su L Cassidy L Liu G Christiani DC. Genome-wide DNA methylation profiling of cell-free serum DNA in esophageal adenocarcinoma and Barrett esophagus. Neoplasia (2012) 14:29–33. 10.1593/neo.111626 22355271 PMC3281939

[36] Martincorena I Roshan A Gerstung M Ellis P Van Loo P McLaren S High burden and perspective positive selection of somatic mutations in normal human skin. Science (2015) 348:880–6. 10.1126/science.aaa6806 25999502 PMC4471149

[37] Guo S Diep D Plongthongkum N Fung HL Zhang K Zhan K. Identification of methylation haplotype blocks aids in deconvolution of heterogeneous tissue samples and tumor tissue-of-origin mapping from plasma DNA. Nat Genet (2017) 49(4):635–42. 10.1038/ng.3805 28263317 PMC5374016

[38] De la Cruz FF Corcoran RB Methylation in cell-free DNA for early cancer detection. Ann Oncol (2018) 29(6):1351–3. 10.1093/annonc/mdy134 29668834 PMC6005066

[39] Tie J Wang Y Tomasetti C Li L Springer S Kinde I Circulating tumor DNA analysis detects minimal residual disease and predicts recurrence in patients with stage II colon cancer. Sci Transl Med (2016) 8:346ra92. 10.1126/scitranslmed.aaf6219 27384348 PMC5346159

[40] Reinert T Schøler LV Thomsen R Tobiasen H Vang S Nordentoft I Analysis of circulating tumour DNA to monitor disease burden following colorectal cancer surgery. Gut (2016) 65:625–34. 10.1136/gutjnl-2014-308859 25654990

[41] Diehl F Schmidt K Choti MA Romans K Goodman S Li M Circulating mutant DNA to assess tumor dynamics. Nat Med (2008) 14:985–90. 10.1038/nm.1789 18670422 PMC2820391

[42] Young GP Pedersen SK Mansfield S Murray DH Baker RT Rabbitt P A cross-sectional study comparing a blood test for methylated BCAT1 and IKZF1 tumor-derived DNA with CEA for detection of recurrent colorectal cancer. Cancer Med (2016) 5:2763–72. 10.1002/cam4.868 27726312 PMC5083729

[43] Symonds EL Pedersen SK Murray DH Jedi M Byrne SE Rabbitt P Circulating tumour DNA for monitoring colorectal cancer-a prospective cohort study to assess relationship to tissue methylation, cancer characteristics and surgical resection. Clin Epigenetics (2018) 10:63. 10.1186/s13148-018-0500-5 29796114 PMC5956533

[44] Gao Y Zhang K Xi H Cai A Wu X Cui J Diagnostic and prognostic value of circulating tumor DNA in gastric cancer: a meta-analysis. Oncotarget (2017) 8:6330–40. 10.18632/oncotarget.14064 28009985 PMC5351635

[45] Kim K Shin DG Park MK Baik SH Kim TH Kim S Circulating cell-free DNA as a promising biomarker in patients with gastric cancer: diagnostic validity and significant reduction of ctDNA after surgical resection. Ann Surg Treat Res (2014) 86:136–42. 10.4174/astr.2014.86.3.136 24761422 PMC3994618

[46] Ling ZQ Zhao Y Zhou SL Mao WM. MSH2 promoter hypermethylation in circulating tumor DNA is a valuable predictor of disease-free survival for patients with esophageal squamous cell carcinoma. Eur J Surg Oncol (2012) 38:326–32. 10.1016/j.ejso.2012.01.008 22265839

[47] Kawakami K Brabender J Lord RV Groshen S Greenwald BD Krasna MJ Hypermethylated APC DNA in plasma and prognosis of patients with esophageal adenocarcinoma. J Natl Cancer Inst (2000) 92:1805–11. 10.1093/jnci/92.22.1805 11078757

[48] Diehl F Li M Dressman D He Y Shen D Szabo S Detection and quantification of mutations in the plasma of patients with colorectal tumors. Proc Natl Acad Sci U S A (2005) 102:16368–73. 10.1073/pnas.0507904102 16258065 PMC1283450

[49] Tie J Kinde I Wang Y Wong HL Roebert J Christie M Circulating tumor DNA as an early marker of therapeutic response in patients with metastatic colorectal cancer. Ann Oncol (2015) 26:1715–22. 10.1093/annonc/mdv177 25851626 PMC4511218

[50] Douillard JY Oliner KS Siena S Tabernero J Burkes R Barugel M Panitumumab-FOLFOX4 treatment and RAS mutations in colorectal cancer. N Engl J Med (2013) 369:1023–34. 10.1056/NEJMoa1305275 24024839

[51] Patterson SD Peeters M Siena S Van Cutsem E Humblet Y Van Laethem JL Comprehensive analysis of KRAS and NRAS mutations as predictive biomarkers for single agent panitumumab (pmab) response in a randomized, phase III metastatic colorectal cancer (mCRC) study (20020408). J Clin Oncol (2013) 31:3617 10.1200/jco.2013.31.15_suppl.3617

[52] Van Cutsem E Lenz HJ Köhne CH Heinemann V Tejpar S Melezínek I Fluorouracil, leucovorin, and irinotecan plus cetuximab treatment and RAS mutations in colorectal cancer. J Clin Oncol (2015) 33:692–700. 10.1200/JCO.2014.59.4812 25605843

[53] Peeters M Oliner K Price TJ Cervantes A Sobrero AF Ducreux M Analysis of KRAS/NRAS mutations in a phase III study of panitumumab with FOLFIRI compared with FOLFIRI alone as second-line treatment for metastatic colorectal cancer. Clin Cancer Res (2015) 21:5469–79. 10.1158/1078-0432.CCR-15-0526 26341920

[54] Bokemeyer C Köhne CH Ciardiello F Lenz HJ Heinemann V Klinkhardt U FOLFOX4 plus cetuximab treatment and RAS mutations in colorectal cancer. Eur J Cancer (2015) 51:1243–52. 10.1016/j.ejca.2015.04.007 25937522 PMC7508202

[55] Stintzing S Modest DP Rossius L Lerch MM von Weikersthal LF Decker T FOLFIRI plus cetuximab versus FOLFIRI plus bevacizumab for metastatic colorectal cancer (FIRE-3): a post-hoc analysis of tumour dynamics in the final RAS wild-type subgroup of this randomised open-label phase 3 trial. Lancet Oncol (2016) 17:1426–34. 10.1016/S1470-2045(16)30269-8 27575024

[56] Tabernero J Lenz HJ Siena S Sobrero A Falcone A Ychou M Analysis of circulating DNA and protein biomarkers to predict the clinical activity of regorafenib and assess prognosis in patients with metastatic colorectal cancer: a retrospective, exploratory analysis of the CORRECT trial. Lancet Oncol (2015) 16:937–48. 10.1016/S1470-2045(15)00138-2 26184520 PMC7513622

[57] Schmiegel W Scott RJ Dooley S Lewis W Meldrum CJ Pockney P Blood-based detection of RAS mutations to guide anti-EGFR therapy in colorectal cancer patients: concordance of results from circulating tumor DNA and tissue-based RAS testing. Mol Oncol (2017) 11:208–19. 10.1002/1878-0261.12023 28106345 PMC5527457

[58] Vidal J Muinelo L Dalmases A Jones F Edelstein D Iglesias M Plasma ctDNA RAS mutation analysis for the diagnosis and treatment monitoring of metastatic colorectal cancer patients. Ann Oncol (2017) 28:1325–32. 10.1093/annonc/mdx125 28419195 PMC5834035

[59] Jones FS Edelstein D Wichner K Ross C Stieler K Boehm V Performance of standardized BEAMing platform for detecting RAS mutations in the blood of metastatic colorectal cancer (mCRC) patients. J Clin Oncol (2016) 34:11538 10.1200/JCO.2016.34.15_suppl.11538

[60] Grasselli J Elez E Caratù G Matito J Santos C Macarulla T Concordance of blood- and tumor-based detection of RAS mutations to guide anti-EGFR therapy in metastatic colorectal cancer. Ann Oncol (2017) 28:1294–301. 10.1093/annonc/mdx112 28368441 PMC5834108

[61] Diaz LA Jr Williams RT Wu J Kinde I Hecht JR Berlin J The molecular evolution of acquired resistance to targeted EGFR blockade in colorectal cancers. Nature (2012) 486:537–40. 10.1038/nature11219 22722843 PMC3436069

[62] Siravegna G Mussolin B Buscarino M Corti G Cassingena A Crisafulli G Clonal evolution and resistance to EGFR blockade in the blood of colorectal cancer patients. Nat Med (2015) 21:795–801. 10.1038/nm.3870 26030179 PMC4868598

[63] Mohan S Heitzer E Ulz P Lafer I Lax S Auer M Changes in colorectal carcinoma genomes under anti-EGFR therapy identified by whole-genome plasma DNA sequencing. PLoS Genet (2014) 10:e1004271. 10.1371/journal.pgen.1004271 24676216 PMC3967949

[64] Tran B Kopetz S Tie J Gibbs P Jiang ZQ Lieu CH Impact of BRAF mutation and microsatellite instability on the pattern of metastatic spread and prognosis in metastatic colorectal cancer. Cancer (2011) 117:4623–32. 10.1002/cncr.26086 21456008 PMC4257471

[65] Pietrantonio F Petrelli F Coinu A Di Bartolomeo M Borgonovo K Maggi C Predictive role of BRAF mutations in patients with advanced colorectal cancer receiving cetuximab and panitumumab: a meta-analysis. Eur J Cancer (2015) 51:587–94. 10.1016/j.ejca.2015.01.054 25673558

[66] Rowland A Dias MM Wiese MD Kichenadasse G McKinnon RA Karapetis CS Meta-analysis of BRAF mutation as a predictive biomarker of benefit from anti-EGFR monoclonal antibody therapy for RAS wild-type metastatic colorectal cancer. Br J Cancer (2015) 112:1888–94. 10.1038/bjc.2015.173 25989278 PMC4580381

[67] Prahallad A Sun C Huang S Di Nicolantonio F Salazar R Zecchin D Unresponsiveness of colon cancer to BRAF(V600E) inhibition through feedback activation of EGFR. Nature (2012) 483:100–3. 10.1038/nature10868 22281684

[68] Corcoran RB Ebi H Turke AB Coffee EM Nishino M Cogdill AP EGFR-mediated re-activation of MAPK signaling contributes to insensitivity of BRAF mutant colorectal cancers to RAF inhibition with vemurafenib. Cancer Discov (2012) 2:227–35. 10.1158/2159-8290.CD-11-0341 22448344 PMC3308191

[69] Corcoran RB Andre T Atreya CE Schellens JHM Yoshino T Bendell JC Combined BRAF, EGFR, and MEK inhibition in patients with BRAF V600E-mutant colorectal cancer. Cancer Discov (2018) 8:428–43. 10.1158/2159-8290.CD-17-1226 29431699 PMC5882509

[70] Leto SM Sassi F Catalano I Torri V Migliardi G Zanella ER Sustained inhibition of HER3 and EGFR is necessary to induce regression of HER2-amplified gastrointestinal carcinomas. Clin Cancer Res (2015) 21:5519–31. 10.1158/1078-0432.CCR-14-3066 26296355

[71] Sartore-Bianchi A Trusolino L Martino C Bencardino K Lonardi S Bergamo F Dual-targeted therapy with trastuzumab and lapatinib in treatment-refractory, KRAS codon 12/13 wild-type, HER2-positive metastatic colorectal cancer (HERACLES): a proof-of-concept, multicentre, open-label, phase 2 trial. Lancet Oncol (2016) 17:738–46. 10.1016/S1470-2045(16)00150-9 27108243

[72] Bertotti A Migliardi G Galimi F Sassi F Torti D Isella C A molecularly annotated platform of patient-derived xenografts (“xenopatients”) identifies HER2 as an effective therapeutic target in cetuximab-resistant colorectal cancer. Cancer Discov (2011) 1:508–23. 10.1158/2159-8290.CD-11-0109 22586653

[73] Takegawa N Yonesaka K Sakai K Ueda H Watanabe S Nonagase Y HER2 genomic amplification in circulating tumor DNA from patients with cetuximab-resistant colorectal cancer. Oncotarget (2016) 7:3453–60. 10.18632/oncotarget.6498 26657506 PMC4823119

[74] Hainsworth JD Bernstam FM Swanton C Hurwitz H Spigel DR Sweeney C Targeted therapy for advanced solid tumors on the basis of molecular profiles: results from MyPathway, an open-label, phase IIa multiple basket study. J Clin Oncol (2018) 36:536–42. 10.1200/JCO.2017.75.3780 29320312

[75] Nakamura Y Okamoto W Sawada K Komatsu Y Kato K Taniguchi H TRIUMPH Study: a multicenter Phase II study to evaluate efficacy and safety of combination therapy with trastuzumab and pertuzumab in patients with HER2-positive metastatic colorectal cancer (EPOC1602). Ann Oncol (2017) 28:612Ti 10.1093/annonc/mdx393.137

[76] Bardelli A Corso S Bertotti A Hobor S Valtorta E Siravegna G Amplification of the MET receptor drives resistance to anti-EGFR therapies in colorectal cancer. Cancer Discov (2013) 3:658–73. 10.1158/2159-8290.CD-12-0558 23729478 PMC4078408

[77] Raghav K Morris V Tang C Morelli P Amin HM Chen K MET amplification in metastatic colorectal cancer: an acquired response to EGFR inhibition, not a de novo phenomenon. Oncotarget (2016) 7:54627–31. 10.18632/oncotarget.10559 27421137 PMC5342368

[78] Strickler JH Rushing CN Uronis HE Morse M Blobe GC Zafar Y Phase Ib study of cabozantinib plus panitumumab in KRAS wild-type (WT) metastatic colorectal cancer (mCRC). J Clin Oncol (2016) 34:3548 10.1200/JCO.2016.34.15_suppl.3548

[79] Bang YJ Cutsem EV Feyereislova A Chung HC Shen L Sawaki A Trastuzumab in combination with chemotherapy versus chemotherapy alone for treatment of HER2-positive advanced gastric or gastro-esophageal junction cancer (ToGA): a phase 3, open-label, randomized controlled trial. Lancet (2010) 376:687–97. 10.1016/S0140-6736(10)61121-X 20728210

[80] Gravalos C Jimeno A. Her2 in gastric cancer: a new prognostic factor and a novel therapeutic target. Ann Oncol (2008) 19:1523–9. 10.1093/annonc/mdn169 18441328

[81] Shoda K Masuda K Ichikawa D Arita T Miyakami Y Watanabe M HER2 amplification detected in the circulating DNA of patients with gastric cancer: a retrospective pilot study. Gastric Cancer (2015) 18:698–710. 10.1007/s10120-014-0432-5 25322965

[82] Shoda K Ichikawa D Fujita Y Masuda K Hiramoto H Hamada J Monitoring the HER2 copy number status in circulating tumor DNA by droplet digital PCR in patients with gastric cancer. Gastric Cancer (2017) 20:126–35. 10.1007/s10120-016-0599-z 26874951

[83] Kim ST Banks KC Lee SH Kim K Park JO Park SH Prospective feasibility study for using cell-free circulating tumor DNA-guided therapy in refractory metastatic solid cancers: an interim analysis. JCO Precision Oncol (2017) 1:1–15. 10.1200/PO.16.00059 PMC7446388 32913970

